# Selective regulation of tuft cell-like small cell lung cancer by novel transcriptional co-activators C11orf53 and COLCA2

**DOI:** 10.1038/s41421-022-00470-7

**Published:** 2022-10-18

**Authors:** Chen Zhou, Hui Huang, Yunyi Wang, Erdem Sendinc, Yang Shi

**Affiliations:** 1 Nuffield Department of Medicine, Ludwig Institute for Cancer Research, University of Oxford, Oxford, UK; 2 Division of Newborn Medicine, Boston Children’s Hospital, Harvard Medical School, Boston, MA USA; 3 PhD Program in Biological and Biomedical Sciences, Harvard Medical School, Boston, MA USA

**Keywords:** Chromatin remodelling, Small-cell lung cancer

Dear Editor,

An ideal cancer therapy drug should kill cancer cells while displaying limited toxicities toward normal cells^1^. Therefore, genes essential in cancer but not normal cells are good therapeutic targets. However, while genetic dependencies for many cancer cell lines have been defined through genetic screens, much less was known for normal cells. To overcome this problem, the DepMap project has developed a method to identify genes selectively essential in a subset of cancer cell lines, making them less likely to be in core pathways critical for normal cells. Based on genome-wide CRISPR-based fitness screen data from ~1000 human cancer cell lines^2–4^, DepMap calculates CERES scores to measure the effect of gene knockout on cell fitness (0: non-essential; −1: median of all common essential genes). Next, DepMap calculates a Normality Likelihood Ratio Test (NormLRT) score for each gene. A higher NormLRT score indicates that the distribution of this gene’s CERES scores across cancer cell lines is more deviated from a normal distribution, and the gene may be more selectively essential. Identification of selectively essential genes will not only deepen our understanding of cancer biology but also guide cancer drug development. However, manually sorting out which of them is understudied and warrants investigations is time-consuming and probably impractical.

To prioritize understudied selectively essential genes, we first recalculated the NormLRT scores because they were not directly available. By using a threshold (NormLRT > 125) reported previously^4^, we collected 347 potential selectively essential genes and ranked them by PubMed publication count^5^. In our ranking system, the top nine most understudied genes are *C11orf53*, *C3orf38*, *TMEM164*, *ZNF511*, *KCNK13*, *BEST3*, *CYB561A3*, *C12orf49*, and *COLCA2* (Fig. 1a). In fact, CYB561A3 has only recently been identified as the key lysosomal iron reductase and a novel cancer vulnerability in Burkitt lymphoma^6^. This demonstrated that our strategy can efficiently identify novel cancer therapeutic targets.

**Fig. 1:**
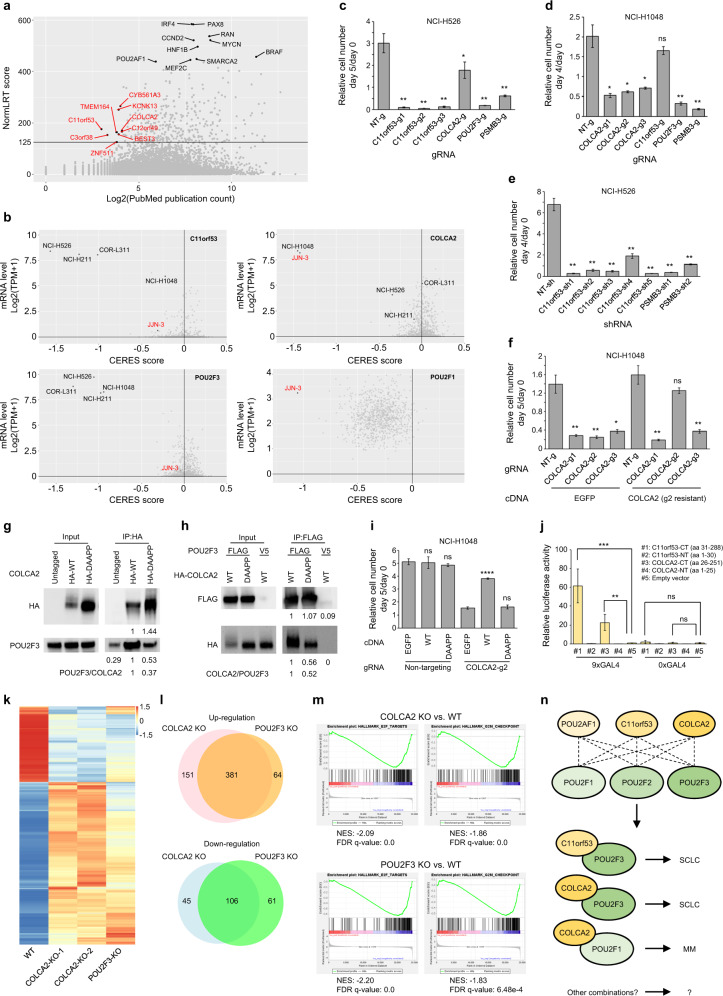
Selective regulation of tuft cell-like SCLC by novel transcriptional co-activators C11orf53 and COLCA2. **a** Scatter plot showing each gene’s NormLRT score vs publication count. **b** Scatter plots showing specific genes’ expression levels vs CERES scores across cancer cell lines. **c** CellTiter-Glo assay of gRNA-treated NCI-H526 cells. PSMB3, a common essential gene, was a positive control. NT nontargeting. *n* = 3. **d** CellTiter-Glo assay of gRNA-treated NCI-H1048 cells. *n* = 3. **e** CellTiter-Glo assay of shRNA-treated NCI-H526 cells. *n* = 3. **f** CellTiter-Glo assay of gRNA-treated EGFP/COLCA2-expressing NCI-H1048 cells. *n* = 3. Statistical analysis was performed within each cDNA group. **g** Immunoprecipitation-western blot of HEK293T cells expressing HA-COLCA2 (WT or mutant where VKELL in the motif is mutated to DAAPP) and POU2F3. Data were quantified. **h** Immunoprecipitation-western blot of HEK293T cells expressing FLAG-POU2F3 and HA-COLCA2-WT/DAAPP. Data were quantified. **i** CellTiter-Glo assay of gRNA-treated EGFP/COLCA2-WT/DAAPP-expressing NCI-H1048 cells. *n* = 3. Statistical analysis was performed within each gRNA group. **j** Luciferase reporter assay of N-terminal (NT) and C-terminal (CT) domains of C11orf53/COLCA2 fused to GAL4 DNA-binding domain. Reporter gene was driven by a minimal promoter downstream of 9×/0×GAL4-binding sites. *n* = 6. **k** Heatmap showing different samples’ normalized expression levels of up/downregulated genes in NCI-H1048 cells upon COLCA2 knockout (KO). **l** Venn diagrams showing the overlap of up/downregulated genes in NCI-H1048 cells upon COLCA2/POU2F3 KO. **m** GSEA analysis of transcriptomic changes in NCI-H1048 cells upon COLCA2/POU2F3 KO. **n** Model. Two-tailed unpaired Student’s *t*-test was performed for all statistical analysis. *P < 0.05; **P < 0.01; ***P < 0.001; ****P < 0.0001.

We subsequently focused on C11orf53 and COLCA2 because of the significant CERES scores in their dependent cell lines where they are highly expressed, suggesting strong growth phenotypes (Fig. 1b). Remarkably, the co-dependencies precalculated by DepMap revealed that all three C11orf53-dependent cell lines are DNA-binding transcription factor POU2F3-dependent small cell lung cancer (SCLC) lines, and one of the only two COLCA2-dependent cell lines is also a POU2F3-dependent SCLC line. Specifically, C11orf53 is essential in SCLC cell lines COR-L311, NCI-H526, and NCI-H211, while COLCA2 is essential in NCI-H1048. This mutually exclusive requirement is likely due to their differential expression. The POU2F3-dependent SCLC was previously reported as a tuft cell-like variant^7^. However, the mechanistic details of how POU2F3 drives the expression of the tuft cell-specific program remain unclear. Based on our analysis, we hypothesized that C11orf53/COLCA2 and POU2F3 may work in the same pathway to regulate the growth of tuft cell-like SCLC.

Consistent with the DepMap data, three different C11orf53 gRNAs significantly decreased NCI-H526 cell growth (Fig. 1c). The COLCA2 gRNA also decreased NCI-H526 cell growth but to a lesser extent. Similarly, three different COLCA2 gRNAs, but not the C11orf53 gRNA, significantly decreased the growth of NCI-H1048 cells (Fig. 1d). Cas9 has been shown to cause cell toxicity when generating DNA double-stranded breaks at loci with high copy numbers^8,9^. Since copy number increase was observed at *C11orf53* locus in NCI-H526 cells (Supplementary Fig. S1), we wished to rule out this possibility. Consistent with the CRISPR approach, five different C11orf53 shRNAs caused growth defects of NCI-H526 cells (Fig. 1e), suggesting that the growth defect was due to C11orf53 loss. Similar results were seen in COR-L311 cells (Supplementary Fig. S2). In addition, the growth defect of COLCA2 gRNA-treated NCI-H1048 cells was rescued by a gRNA-resistant COLCA2 transgene (Fig. 1f). These findings suggest that C11orf53/COLCA2 is required for SCLC growth.

We next investigated the molecular functions of C11orf53 and COLCA2 by domain search through Pfam^10^ (Supplementary Fig. S3). Interestingly, C11orf53 and COLCA2 share an N-terminal motif, (R/K)xYQGVRVKxxVK(D/E)LLxx(K/R)R, with the transcriptional co-activator POU2AF1 (Supplementary Fig. S4). This motif mediates physical interactions of POU2AF1 with the highly conserved POU-specific domains (Supplementary Fig. S5) in POU domain class 2 family of DNA-binding transcription factors, POU2F1 and POU2F2^11,12^. As discussed, POU2F3, the third member of this family, is essential for all four C11orf53/COLCA2-dependent SCLC cell lines in DepMap (Fig. 1b), leading to our hypothesis that C11orf53 and COLCA2 may act as co-activators of POU2F3 and regulate transcription of genes critical for tuft cell-like SCLC.

Our co-activator hypothesis predicts that C11orf53 and COLCA2 physically interact with POU2F3. Indeed, co-immunoprecipitation detected physical interactions between COLCA2 and POU2F3 (Fig. 1g, h). Furthermore, COLCA2 carrying mutations in the predicted interaction motif showed a reduced interaction with POU2F3 (Fig. 1g, h) and failed to rescue the growth defect caused by COLCA2 loss (Fig. 1i), indicating the importance of the physical interaction. As co-activators, COLCA2 and C11or53 are also predicted to carry transcriptional activation domains. Indeed, when fused to the GAL4 DNA-binding domain, the C-terminal region of C11orf53 (aa 31–288) or COLCA2 (aa 26–251) activated transcription of the luciferase reporter gene in a GAL4-binding sites-dependent manner (Fig. 1j). In addition, ectopic expression of POU2F3 with C11orf53/COLCA2 in HEK293T cells activated expression of AVIL (Supplementary Fig. S6), a known POU2F3 direct target in NCI-H1048 cells^7^. In contrast, expression of each factor individually or co-expression of mutant C11orf53/COLCA2 with POU2F3 did not activate AVIL expression. Collectively, these findings identify COLCA2 (and likely C11orf53) as a co-activator for POU2F3 and demonstrate that their physical interaction is critical for the growth of SCLC.

To explore the molecular basis underlying the growth defect of COLCA2-deficient NCI-H1048 cells, we performed RNA-sequencing. Consistent with our hypothesis, most genes with significant changes upon COLCA2 knockout (adjusted *P*-value < 0.05) showed similar changes upon POU2F3 knockout (Fig. 1k). In addition, there was a large overlap between the up/downregulated genes (adjusted *P*-value < 0.05, fold change > 2) upon COLCA2/POU2F3 knockout (Fig. 1l). Although more genes were upregulated than downregulated upon COLCA2 knockout, the POU2F3 DNA-binding motif is more enriched in the promoters of downregulated genes (Supplementary Fig. S7), suggesting that the upregulated genes are probably indirectly regulated. POU2F3 was reported to drive the expression of tuft cell markers^7^. In accordance, COLCA2 knockout also downregulated tuft cell markers (Supplementary Fig. S8). Importantly, the downregulated genes upon COLCA2/POU2F3 knockout were significantly enriched in cell cycle-related pathways (Fig. 1m), many of which are known positive regulators of the cell cycle, including CDC25A, CENPE, and KIF15 (Supplementary Fig. S9), which may explain the growth defect. These observations provide further functional evidence supporting the model that COLCA2 functions as a co-activator for POU2F3.

In summary, we used PubMed publication count to prioritize understudied potential cancer therapeutic targets and identified C11orf53 and COLCA2 as novel vulnerabilities in tuft cell-like SCLC. We provided further biochemical and functional data demonstrating that COLCA2 (and likely C11orf53) functions as a co-activator for POU2F3 to drive the transcriptional program important for tuft cell-like SCLC. The highly selective nature of these co-activators in cancer coupled with the reports that *Colca2*^–/–^ mice and *C11orf53*^–/–^ mice are viable^13,14^ suggest that disrupting the interactions between these co-activators and POU2F3 could be a viable therapeutic strategy with minimal toxicities. While our work was ongoing, two papers, one in *bioRxiv*^15^ and the other in *Nature*^14^ appeared online in which the authors also investigated the role of C11orf53 (renamed as POU2AF2)^15^ and both C11orf53 (renamed as POU2AF2/OCA-T1) and COLCA2 (renamed as POU2AF3/OCA-T2)^14^ in SCLC, and our conclusion is essentially the same as those reached by these investigators. We noticed that in addition to SCLC, COLCA2 is also essential in a multiple myeloma cell line, JJN-3, where POU2F1 instead of POU2F3, is highly expressed and essential (Fig. 1b), suggesting that COLCA2 may work as a co-activator for POU2F1 to promote multiple myeloma. Based on the findings discussed above, we propose that POU2AF1/C11orf53/COLCA2 are a family of co-activators for POU2F1/2/3 to regulate SCLC and possibly additional cancers such as multiple myeloma (Fig. 1n). How the co-activators are paired with these transcription factors is likely dictated by their relative expression levels. We further noticed that C11orf53 and COLCA2 are overexpressed in a subset of tumor samples across different cancers (Supplementary Fig. S10), suggesting that these two genes could have roles in other cancers.

## Supplementary information

Supplementary information
