# Supplementary material for: Selective regulation of tuft cell-like small cell lung cancer by novel transcriptional co-activators C11orf53 and COLCA2
Source: Cell Discov. 2022 Oct 18;8:112. doi: 10.1038/s41421-022-00470-7 (PMC9576728; doi:10.1038/s41421-022-00470-7)
Supplement: Supplementary file 1 — Supplementary information [file 41421_2022_470_MOESM1_ESM.pdf]

Cell Line	C11orf53 relative copy number<br>Log2(relative ploidy + 1)
NCI-H1048	1.05
NCI-H211	1.15
COR-L311	2.45
NCI-H526	1.77

Supplementary Fig. S1 C11orf53 relative copy number in tuft cell-like SCLC cell lines. The copy number data was from DepMap. Relative copy number = 1 means the copy number of this gene is the same as most of the rest of the genome.

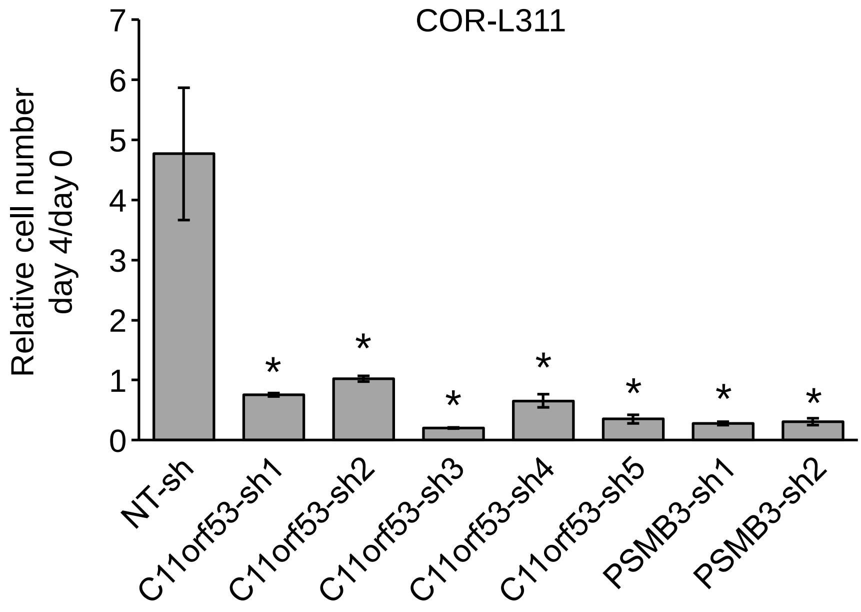

Supplementary Fig. S2 C11orf53 knock-down decreased growth of COR-L311 cells. CellTiter-Glo assay result of COR-L311 cells infected with different shRNA lentiviruses. COR-L311 cells were infected with shRNA lentiviruses and 2 days later, the same number of cells were seeded in 96-well plate. CellTiter-Glo assay was performed on day 0 and day 4. NT: non-targeting. n=3, two-tailed unpaired Student's t test. *P < 0.05.

Human C11orf53

Family	Description	Entry type	Clan	Envelope		Alignment		HMM		HMM length	Bit score	E-value
				Start	End	Start	End	From	To			
PD-C2-AF1	POU domain, class 2, associating factor 1 (shorten)	Domain	n/a	2	129	9	79	**9**	**70**	249	19.8	0.00041

```
#HMM       sprpyqgvrvkepvkellkrkr.gsvssg....aav..aattvvlphqplas.ystvgqscl.dvevaast
#MATCH     s r yqgvrvk  vk+ll  kr g+ s++     +      +  v +p  p++s y  v +s l d+++  s
#PP        6799**************99994555443111122200223466676666544444444433266665555
#SEQ       SKRVYQGVRVKHTVKDLLAEKRsGQTSNSrlngSVSssQSPFVQMPGSPVTSgYYGVRRSFLsDSDFHNSK
```

Human COLCA2

Family	Description	Entry type	Clan	Envelope		Alignment		HMM		HMM length	Bit score	E-value
				Start	End	Start	End	From	To			
PD-C2-AF1	POU domain, class 2, associating factor ...	Domain	n/a	1	85	4	38	**9**	**42**	249	22.2	7.7e-05

```
#HMM       sprpyqgvrvkepvkellkrkrgsv.ssgaavaat
#MATCH     +p+ yqgvrvk  vkell+++r +  +sg + +
#PP        799****************9999864555554443
#SEQ       KPKVYQGVRVKITVKELLQQRRAHQaASGGTRSGG
```

Supplementary Fig. S3 Pfam protein domain prediction result. Pfam was used to predict protein domains in human C11orf53 and COLCA2.

1. Human_POU2AF1
2. Human_C11orf53
3. Human_COLCA2

```
1    MLWQKPTAPEQAPA--PARPYQGVRVKEPVKELLRRKRGHASS-----GAAPAPTAVVL--PHQPLATYT   61
2    --------MESVPGDYSKRVYQGVRVKHTVKDLLAEKRSGQTSNSRLNGSVSSSQSPFVQMPGSPVTSGY   62
3    --------MSEKP-----KVYQGVRVKITVKELLQQRRAHQAASG---GTRSGGSSVHLSDPVAPSSAGL   54

1    TVGPSCL----------DMEGSVSAVTEEAAL---CAGWL-SQPTPATLQPLAPWTPYTEYVPHEAVSCP   117
2    YGVRRSFLSDSDFHNSKQFSNDVYTSSVGKPF--PCESSA-GQSHAALLEPYFPQEPYGDYRPPAL--TP   127
3    YFEPEPISSTPNYLQRGEFSSCVSCEENSSCLDQIFDSYLQTEMHPEPLLNSTQSAPH--HFPDSFQATP   122

1    YSADMY-VQPVCPSYTVVGPSSVLTYASPPLITNVTTRSSATPA-------VGPPLEGPEHQA-------   172
2    NAGSLFSASPLPPLLPPPFPGDPAHFLFRDSWEQTLPDGLSQPDPVSADALLTLPPSTSCLSQLESGSIA   197
3    FCFNQS-LIPGSPSNSSILSGSLDYSYSPVQLPSYAPENYNSPASLDTRT-CGYPPEDHSYQHLSS----   186

1    PLTYFPWPQPLSTLPTSTLQY---------QPPAPALPGPQFVQLPISIPEPVLQDMEDPRRAASSLTID   233
2    QHRGSSWGSSLAGAQSYSLHALEDLHHTPGYPTPPPYPFTPFMTVSNDLP-PKVGPLSPDEEADTGSLHD   266
3    HAQYSCFSSATTSICYCASCEAEDLDAL--QAAEYFYPSTDCVDFA-------------PSAAATSDFYK   241

1    KLLLEEEDSDAYALNHTLSVEGF   256
2    PSPWVKEDGSI-AWGSYECRRAY   288
3    R----ETNCDI-CYS--------   251
```

Supplementary Fig. S4 Protein sequence alignment result of human POU2AF1/C11orf53/COLCA2. Protein sequence alignment identifies a shared, conserved sequence motif (highlighted) at the N-terminal regions of human POU2AF1, C11orf53 and COLCA2.

1. Human_POU2F1
2. Human_POU2F2
3. Human_POU2F3

```
1   ------------MNNPSETSKPSMESGDG-NTGTQTNGLDFQKQPVPVGGAISTAQAQAFLGHLHQVQLA  57
2   MVHSSMGAPEIRMSKPLEAEKQGLDSPSE-HTDTERNGPDTNHQ---------------------NPQNK  48
3   MVNLESMHTDIKMSGDVADSTDARSTLSQVEPGNDRNGLDFNRQ--------------------------  44

1   GTSLQAAAQSLNVQSKSNEESGDSQQPSQPSQQPSVQAAIPQTQLMLAGGQITGLTLTPAQQQLLLQQAQ  127
2   TSPFSVSPTGPSTKIKAEDPSGDS-APAAPLPPQPAQPHLPQAQLMLTGSQLAG----------------  101
3   --------------IKTEDLS-DSLQQTLSHRP----CHLSQGPAMMSGNQMSGLNASP-----------  84

1   AQAQLLAAAVQQHSASQQHSAAGATISASAATPMTQIPLSQPIQIAQDLQQLQQLQQQNLNLQQFVLVHP  197
2   -----------------------------------------------DIQQLLQLQQ--------LVLVP  116
3   ---------------------------------------------CQDMASLHPLQQ--------LVLVP  101

1   TTNLQP-AQFIISQTPQGQQGLLQAQNLLTQLPQQSQANLLQSQPSITLTSQ----PATPTRTIAATPIQ  262
2   GHHLQPPAQFLLPQAQQSQPGLLPTPNLF-QLPQQTQGALLTSQPRAGLPTQAVTRPTLP------DPHL  179
3   G-HLQSVSQFLLSQTQPGQQGL--QPNLL-PFPQQ-QSGLLLPQTGPGLASQAFGHPGLPGSSL--EPHL  164

1   TLPQSQSTPKRIDTPS-LEEPSDLEELEQFAKTFKQRRIKLGFTQGDVGLAMGKLYGNDFSQTTISRFEA  331
2   SHPQP---PKCLEPPSHPEEPSDLEELEQFARTFKQRRIKLGFTQGDVGLAMGKLYGNDFSQTTISRFEA  246
3   EASQHLPVPKHLPSSGGADEPSDLEELEKFAKTFKQRRIKLGFTQGDVGLAMGKLYGNDFSQTTISRFEA  234

1   LNLSFKNMCKLKPLLEKWLNDAENLSSDSSLSSPSALNSP--GIEGL-SRRRKKRTSIETNIRVALEKSF  398
2   LNLSFKNMCKLKPLLEKWLNDAETMSVDSSLPSPNQLSSPSLGFDGLPGRRRKKRTSIETNVRFALEKSF  316
3   LNLSFKNMCKLKPLLEKWLNDAESSPSDPSVSTPS--SYP--SLSEVFGRKRKKRTSIETNIRLTLEKRF  300

1   LENQKPTSEEITMIADQLNMEKEVIRVWFCNRRQKEKRINPPSSGGTSSSPIKAIFPSPTSLVATTPSLV  468
2   LANQKPTSEEILLIAEQLHMEKEVIRVWFCNRRQKEKRINPCSAA--------PMLPSPGKPASYSPHMV  378
3   QDNPKPSSEEISMIAEQLSMEKEVVRVWFCNRRQKEKRINCPVAT-------------PIKPPVYNSRLV  357

1   TSSAATTLTVSPVLPLTSAAVTNLSVTGTSDTTSNNTATVISTAPPASSAVTSPSLSPSPSASASTSEAS  538
2   TPQGGAG-----TLPLSQA---------------------------------------------------  392
3   SPSGSLG-------PLSVPPV-------------------------------------------------  371

1   SASETSTTQTTSTPLSSPLGTSQVMVTASGLQTAAAAALQGAAQLPANASLAAMAAAAGLNPSLMAPSQF  608
2   ----SSSLSTTVTTLSSAVGT-------------------------------------------LHPSRT  415
3   ----HSTMPGTVTSSCSP----------------------------------------------------  385
```

Supplementary Fig. S5 Protein sequence alignment result of human POU2F1/POU2F2/POU2F3. The conserved POU-specific domain is highlighted.

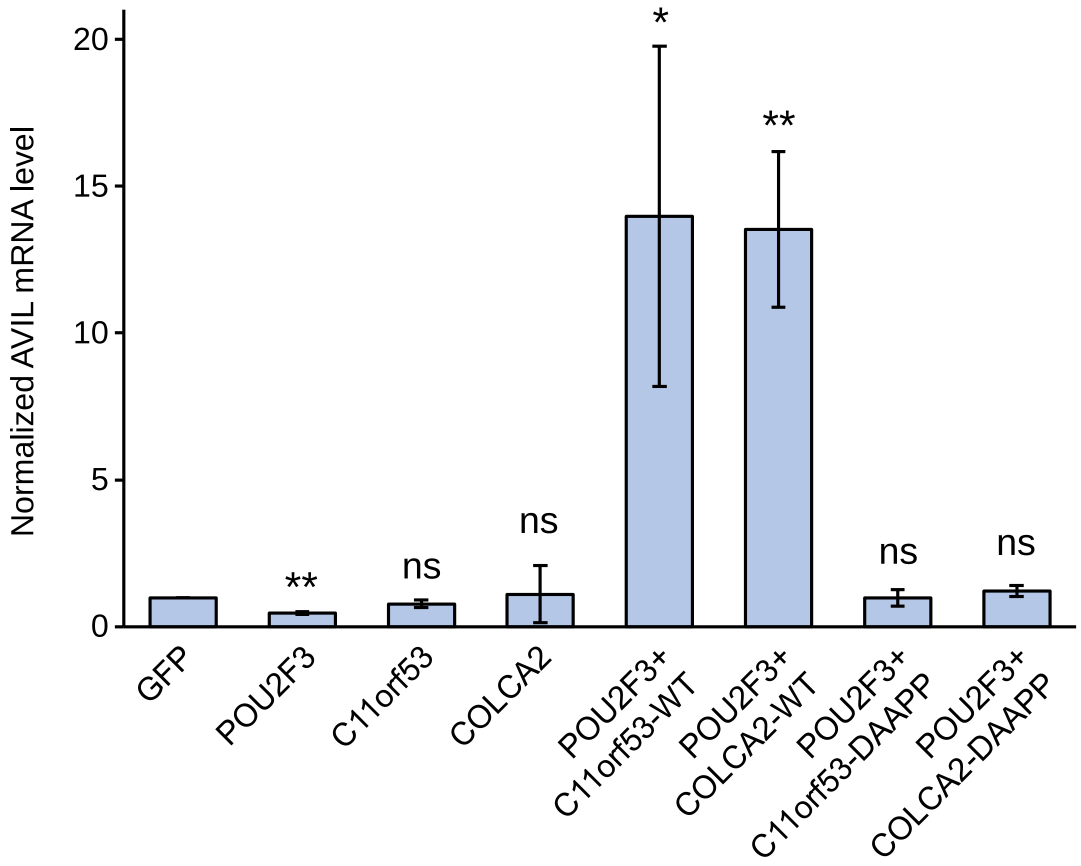

Supplementary Fig. S6 Normalized AVIL mRNA levels in HEK293T cells transfected with different DNA constructs. HEK293T cells were transfected with different expression constructs. 48h later, cells were collected for RT-qPCR. The data was normalized to GAPDH and the GFP control sample. n=3, one-tailed unpaired Student's t test. *P < 0.05, **P<0.01.

Motif	Factor name	p-value
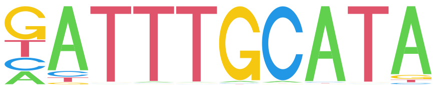	POU2F3	1e-4
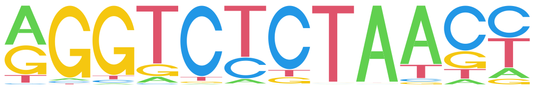	PRDM14	1e-3
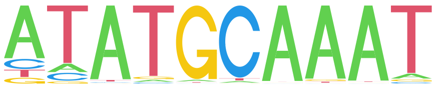	POU2F2	1e-3
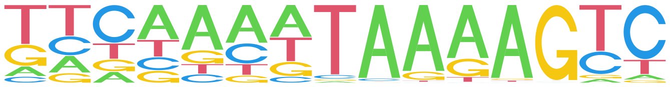	MYNN	1e-2
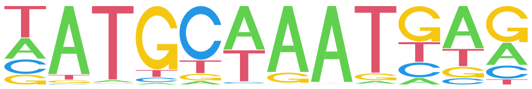	POU3F1	1e-2

Supplementary Fig. S7 Motifs more enriched in the promoter regions of down-regulated than up-regulated genes upon COLCA2 knockout in NCI-H1048 cells. Homer was used to find the motifs more enriched in the promoter regions (TSS +/- 1kb) of down-regulated than up-regulated genes.

Gene name	Log2 fold change (COLCA2 KO vs. WT)	Adjusted p-value
SOX9	-1.04	6.28e-4
NREP	-1.86	2.00e-62
LRMP	-2.33	6.10e-12
GFI1B	-1.79	1.08e-10
AVIL	-2.33	4.82e-34

Supplementary Fig. S8 Gene expression changes of tuft cell markers in COLCA2 KO vs. WT NCI-H1048 cells .

Gene name	Log2 fold change (COLCA2 KO vs. WT)	Adjusted p-value
CDC25A	-0.68	1.39e-2
CENPE	-0.88	3.20e-5
KIF15	-0.63	8.53e-3
KNL1	-0.72	3.33e-4
ESPL1	-0.69	5.38e-5
PTTG1	-0.71	2.88e-6

Supplementary Fig. S9 Gene expression changes of cell cycle regulators in COLCA2 KO vs. WT NCI-H1048 cells .

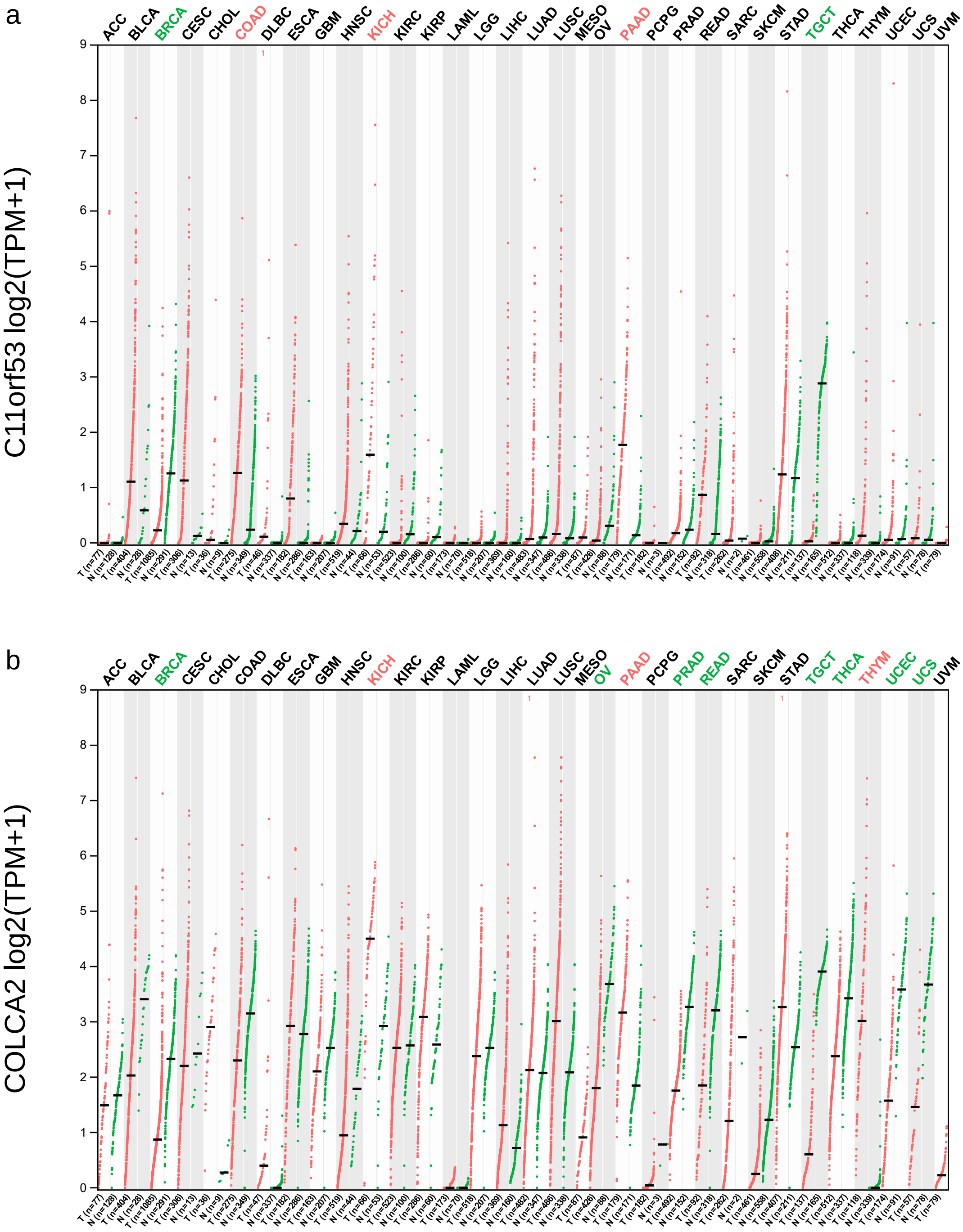

Supplementary Fig. S10 Gene expression level of C11orf53/COLCA2 in different tumor and normal samples. a Data from GEPIA showing that C11orf53 is highly expressed in a subset of tumor samples across different cancers. **b** Data from GEPIA showing that COLCA2 is highly expressed in a subset of tumor samples across different cancers. Red dots are tumor samples and green dots are normal samples.

Supplementary Methods

Cell lines

HEK293T, NCI-H1048 and NCI-H526 cells were obtained from ATCC. COR-L311 cells were obtained from ECACC. HEK293T cells were cultured in DMEM supplemented with 10% FBS and 1% penicillin/streptomycin. NCI-H1048 cells were cultured in DMEM/F12 supplemented with 5% FBS, 0.005 mg/mL insulin, 0.01 mg/mL transferrin, 30 nM sodium selenite, 10 nM hydrocortisone and 10 nM beta-estradiol. NCI-H526 cells and COR-L311 cells were cultured in RPMI1640 supplemented with 10% FBS. All the cells were cultured in a 5% CO2 incubator at 37ºC.

Antibodies

Antibodies used in this study include HA (Cell Signaling Technology #14031S, 1:1000 for western blot), FLAG (Sigma Aldrich #F1804, 1:1000 for western blot) and POU2F3 (Sigma Aldrich #HPA019562, 1:500 for western blot).

Gene depletion by CRISPR/Cas9

The gRNA oligos, with sequences for their respective target genes listed below, were annealed and cloned into a lentiCRISPR v2 lentiviral vector. lentiCRISPR v2 was a gift from Feng Zhang (Addgene #52961)[1]. Lentivirus carrying lentiCRISPR v2 plasmid was produced by co-transfecting HEK293T cells with two packaging plasmids, psPAX2 (Addgene #12260) and pMD2.G (Addgene #12259, both were gifts from Didier Trono), and by harvesting viral supernatant after 48 h by passing through a 0.45 um filter. Collected lentivirus was used directly to infect NCI-H1048, NCI-H526 and COR-L311 cells with the addition of 4 ug/ml polybrene by centrifugation at 550 g for 30 min. 48h later, the infected cells were selected with puromycin for 4-5 days before being used for subsequent assays.

gRNA name	Target sequence
C11orf53_gRNA1	AGTCTCCGTAGGGCTCCTGG
C11orf53_gRNA2	GGAAGTGAGCTGGGTCTCCG
C11orf53_gRNA3	ACGTCTACACCTCCAGCGTG
COLCA2_gRNA1	CTTTGTGTGGAATTGAGCAA
COLCA2_gRNA2	TCTGGTGTCCAGAGAAGCAG
COLCA2_gRNA3	ATGGTGTGGAGCACTTTGTG
POU2F3_gRNA	GACCAACATCCGCCTGACTC
PSMB3_gRNA	ATGTACAGCCGGTCACCCAT
Non-targeting_gRNA	ATCGTTTCCGCTTAACGGCG

Expression plasmid construction

COLCA2-gRNA2-resistant COLCA2 cDNA was amplified from cDNA library reverse transcribed from RNA extracted from NCI-H1048 cells. C11orf53 cDNA was amplified from cDNA library reverse transcribed from RNA extracted from NCI-H526 cells. EGFP cDNA, C11orf53 cDNA, untagged/HA-tagged WT/mutant (VKELL mutated to DAAPP) COLCA2-gRNA2-resistant COLCA2 cDNA were cloned into pCMV-blasticidin lentiviral vector by using NEBuilder HiFi DNA assembly. LentiV-Neo-POU2F3 was a gift from Christopher Vakoc (Addgene #122175)[2]. V5- or FLAG-tagged POU2F3 were cloned into pCMV-puromycin lentiviral vector by using NEBuilder HiFi DNA assembly.

Stable cell line generation

Lentivirus derived from pCMV-blasticidin plasmid expressing WT or mutant COLCA2 and EGFP was produced by co-transfecting HEK293T cells with two packaging plasmids (psPAX2 and pMD2.G) and by harvesting viral supernatant after 48 h by passing through a 0.45 um filter. Collected lentivirus was used directly to infect NCI-H1048 cells with the addition of 4 ug/ml polybrene by centrifugation at 550 g for 30 min. 48h later, the infected cells were selected with 4 ug/mL blasticidin for 4-5 days before being used for subsequent assays.

Gene knockdown by shRNA

The shRNA oligos, with their target sequences listed below, were annealed and cloned into a pLKO.1-Puromycin lentiviral vector (Addgene #10878, a gift from David Root)[3]. Lentivirus carrying pLKO.1 plasmid was produced by co-transfecting HEK293T cells with two packaging plasmids (psPAX2 and pMD2.G) and by harvesting viral supernatant after 48 h by passing through a 0.45 um filter. Collected lentivirus was used directly to infect NCI-H526 and COR-L311 cells with the addition of 4 ug/ml polybrene by centrifugation at 550 g for 30 min. 48h later, the infected cells were used for subsequent assays.

shRNA name	Target sequence
C11orf53_shRNA1	TAACTTGAGGTGTATTCCAAA
C11orf53_shRNA2	CTTTCTTATCTGACTCAGACT
C11orf53_shRNA3	GTGAAAGAAGATGGGAGTATT
C11orf53_shRNA4	CTTATCTGACTCAGACTTCCA
C11orf53_shRNA5	CGGTGTCAGAAGATCTTTCTT
PSMB3_shRNA1	GTCGGCAGATCAAACCTTATA
PSMB3_shRNA2	CTGTATGAGTTGAAGGAAGGT
Non-targeting_shRNA	CTTCGAAATGTCCGTTCGGTT

Cell growth assay

6000 NCI-H1048/NCI-H526/COR-L311 cells were seeded in each well of 96-well plate for cell growth assay in 100 uL volume. 4-5 days later, relative cell number was assessed by using the CellTiter-Glo luminescent assay kit (Promega #G7572) according to the manufacturer’s instructions.

Immunoprecipitation (IP)

HEK293T cells in a 10 cm dish were transfected with the indicated plasmids (10 ug each), and harvested 48 h post-transfection. Cell pellets were lysed for 30 min on ice in IP lysis buffer (20 mM Tris-HCl pH8.0, 150 mM NaCl, 1% Triton X-100, 1.5 mM $MgCl_2$) supplemented with Complete EDTA-free protease inhibitor cocktail (Sigma-Aldrich, cat#5892791001) and PhosSTOP (Sigma-Aldrich, cat#04906837001). Protein lysates were cleared by 10 min centrifugation to pellet cell debris. Cleared protein lysates were incubated with anti-HA magnetic beads (Thermo Fisher Scientific, cat#88837) or anti-FLAG magnetic beads (Thermo Fisher Scientific, cat#A36798) overnight at 4 °C. Immunoprecipitants were then washed three times with the IP lysis buffer and eluted using 100 uL 2x SDS loading buffer.

GAL4 dual-luciferase reporter assay

cDNA fragments encoding the C-terminal and N-terminal domains of C11orf53 and COLCA2 were cloned into the pFN26A (Cat#E1380, Promega) vector by Gibson Assembly (Cat#E2611L, NEB). C11orf53 N-terminal: aa 1-30. C11orf53 C-terminal: aa 31-288. COLCA2 N-terminal: aa 1-25. COLCA2 C-terminal: aa 26-251. The plasmids were then co-transfected with the pGL4.35[luc2P/9XGAL4 UAS/Hygro] vector (Cat#E1370, Promega) or the version without GAL4 binding sites into HEK293T cells. Specifically, 50 ng reporter plasmids was co-transfected with 100 ng pGL4.35 vector/the version without GAL4 binding sites into 10,000 HEK293T cells in each well of 96-well plate by lipofectamine 2000 (Cat#11668019, Invitrogen). Luciferase activity was measured by dual-luciferase reporter assay kit (Cat#E1910, Promega) 48 hours after transfection following the manufacturer's instructions.

RNA-seq

Total RNA was extracted by using TRIzol/chloroform extraction method and further treated with DNase by using RNA Clean & Concentrator (Zymo Research #R1014). Poly(A)+ RNA was selected by using the NEBNext® Poly(A) mRNA Magnetic Isolation Module (NEB #E7490S). RNA-seq libraries were prepared according to the manual for NEBNext® Ultra™ II RNA Library Prep Kit (NEB #E7770L) for Illumina and sequenced on a NextSeq 2000 set at 50 PE mode.

RT-qPCR

The extracted RNA was reversely transcribed into cDNA using the PrimeScript™ RT Master Mix (Perfect Real Time) (Takara #RR036A) according to the manufacturer's instructions. The obtained cDNA samples were diluted and used for real-time quantitative PCR (RT-qPCR). Power SYBR green PCR master mix (Applied Biosystems #4367659) and gene specific primers with sequences (from PrimerBank https://pga.mgh.harvard.edu/primerbank/) listed below were used for PCR amplification and detection on a StepOnePlus real-time PCR system (Applied Biosystems). The RT-qPCR data were normalized to GAPDH and presented as fold changes of gene expression in the test sample compared to the control.

Primer name	Primer sequence
Human AVIL forward	GGTCCAGTACCATGAGTCAGA
Human AVIL reverse	CCGCTTCACGTCGTAGGTAT
Human GAPDH forward	GGAGCGAGATCCCTCCAAAAT
Human GAPDH reverse	GGCTGTTGTCATACTTCTCATGG

Identification of selectively essential genes

CRISPR gene effect table containing CERES scores was downloaded from DepMap Public 21Q2 dataset. To calculate NormLRT score[4] for each gene, the distribution of CERES scores across all cell lines were fitted with both a normal distribution and a skew normal distribution. NormLRT score = 2*(log likelihood ratio of the fitted skew normal distribution – log likelihood ratio of the fitted normal distribution). PubMed publication count was derived from gene2pubmed file available on NCBI FTP.

Data analysis

For RNA-seq data, read counts for genes were determined by using Salmon and differential gene expression analysis was done by using DESeq2. Motif analysis was performed with Homer. Tumor sample data was from GEPIA2 portal (http://gepia2.cancer-pku.cn/#index)[5].

Data availability

All genomic datasets are available on GEO database with GSE212912.

References

1. Sanjana, N. E., Shalem, O. & Zhang, F. Improved vectors and genome-wide libraries for CRISPR screening. *Nat. Methods* **11**, 783–784 (2014).

2. Huang, Y.-H. *et al.* POU2F3 is a master regulator of a tuft cell-like variant of small cell lung cancer. *Genes Dev.* **32**, 915–928 (2018).

3. Moffat, J. *et al.* A Lentiviral RNAi Library for Human and Mouse Genes Applied to an Arrayed Viral High-Content Screen. *Cell* **124**, 1283–1298 (2006).

4. McDonald, E. R. *et al.* Project DRIVE: A Compendium of Cancer Dependencies and Synthetic Lethal Relationships Uncovered by Large-Scale, Deep RNAi Screening. *Cell* **170**, 577-592.e10 (2017).

5. Tang, Z., Kang, B., Li, C., Chen, T. & Zhang, Z. GEPIA2: an enhanced web server for large-scale expression profiling and interactive analysis. *Nucleic Acids Res.* **47**, W556–W560 (2019).
